# Hyperspectral Imagery Super-Resolution by Compressive Sensing Inspired Dictionary Learning and Spatial-Spectral Regularization

**DOI:** 10.3390/s150102041

**Published:** 2015-01-19

**Authors:** Wei Huang, Liang Xiao, Hongyi Liu, Zhihui Wei

**Affiliations:** 1 School of Computer Science and Engineering, Nanjing University of Science & Technology, Nanjing 210094, China; E-Mails: hnhw235@163.com (W.H.); gswei@njust.edu.cn (Z.W.); 2 School of Science, Nanjing University of Science & Technology, Nanjing 210094, China; E-Mail: hyliu@njust.edu.cn

**Keywords:** compressive sensing, dictionary learning, super-resolution, hyperspectral image, spectral similarity, sparse representation

## Abstract

Due to the instrumental and imaging optics limitations, it is difficult to acquire high spatial resolution hyperspectral imagery (HSI). Super-resolution (SR) imagery aims at inferring high quality images of a given scene from degraded versions of the same scene. This paper proposes a novel hyperspectral imagery super-resolution (HSI-SR) method via dictionary learning and spatial-spectral regularization. The main contributions of this paper are twofold. First, inspired by the compressive sensing (CS) framework, for learning the high resolution dictionary, we encourage stronger sparsity on image patches and promote smaller coherence between the learned dictionary and sensing matrix. Thus, a sparsity and incoherence restricted dictionary learning method is proposed to achieve higher efficiency sparse representation. Second, a variational regularization model combing a spatial sparsity regularization term and a new local spectral similarity preserving term is proposed to integrate the spectral and spatial-contextual information of the HSI. Experimental results show that the proposed method can effectively recover spatial information and better preserve spectral information. The high spatial resolution HSI reconstructed by the proposed method outperforms reconstructed results by other well-known methods in terms of both objective measurements and visual evaluation.

## 1. Introduction

Hyperspectral imagery (HSI) has high spectral resolution (containing about 200 spectral band in the visible and infrared wavelength regions, *i.e.*, 400–2500 nm), which is very important for many applications, such as land use analysis, environment studies, military surveillance, and so on. Since hyperspectral sensors have a physical tradeoff between the spatial resolution and spectral resolution, the spatial resolution of HSI is often coarser than that of panchromatic and multispectral images [1]. Practically, a high spatial resolution image can offer more details than a low spatial resolution image due to its higher pixel density. Therefore, in order to benefit from both the spectral and spatial information, image post-processing techniques are employed to improve the spatial resolution of the HSI with less spectral distortions.

To improve the image spatial resolution, the super-resolution (SR) technique is employed, which was firstly proposed by Tsai and Huang [2]. The SR technique is a process of combining one or multiple low-resolution (LR) images of the same scene to produce a high-resolution (HR) image. Due to the ill-posed nature of the SR problem, various regularization methods have been proposed to stabilize this problem. Generally, these methods can be categorized into three classes: interpolation-based methods [3,4], multi-image-based methods [2,5,6], and example-based learning methods for single image SR [7–9]. These methods have achieved great success on gray or color images, but they are not completely suitable for remote sensing images.

The most famous technique for enhancing the spatial resolution of remote sensing images is pan-sharpening. If the corresponding panchromatic images or HR multispectral images are available, pan-sharpening techniques can produce HR HSI by fusing the information of the observed LR HSI and the corresponding panchromatic images or HR multispectral images. The typical methods are the intensity-hue-saturation (IHS) method [10], the principal component analysis (PCA) method [11,12], the wavelet transform (WT) method [13,14], and a variational model for P + XS image fusion [15]. However, these pan-sharpening techniques perform a tradeoff between the spatial resolution and spectral resolution of the HSI.

For the HSI-SR problem, the process of the HSI-SR method not only improves spatial information, but also preserves spectral information. Akgun *et al.* [16] proposed a POCS-based HSI-SR method, which fused the information from multiple observations and spectral bands to improve spatial resolution and reconstruct the spectrum of the observed scene as a combination of a small number of spectral basis functions. In order to estimate motion parameters, Zhang *et al.* [1] presented a maximum *a posteriori* (MAP)-based multi-frame SR method, which utilized principal component analysis (PCA) to reduce the computational load and reconstruct the HR HSI. However, these multi-frame-based SR methods require an accurate registration process that it is a difficult and challenging task [17].

In order to overcome this difficulty, compressive sensing (CS)-based single image super-resolution methods have gained enough attention in the recent years, whereby the high-frequency details of reconstructed HR images can be learned from the HR training images. The CS theory, first proposed by Donoho and Candes *et al.* [18,19], provides a possible way of recovering HR images from the down-sampled LR images under moderate conditions. Yang *et al.* [20] presented a SR method in the CS framework, which needed to train two dictionaries to ensure that the LR and HR image patches have the same coefficients. However, the performance of this method relied heavily on the number of atoms of the dictionaries. In [21,22], the authors only establish one HR dictionary, which needed a few of atoms to resolve the SR problem well, but they fix the linear measurement matrix which is limited only to a scale factor of two. Furthermore, if these methods were directly applied to improve the spatial resolution of every spectral band of HSI individually without considering spectral information, they will destroy the spectral information of the HSI, which is extremely important for the applications of the HSI.

Inspired by these ideas, we propose a novel HSI-SR method via dictionary learning and spatial-spectral regularization. The dictionary for HR HSI is learned from the pre-HR HSI, which is formed from the observed LR HSI by using bicubic interpolation. The advantage of this strategy is that it can improve the adaptively of dictionary and make the method more practical without needing panchromatic images or a HR HSI training database. Furthermore, the HR dictionary is learned with strong sparsity and small coherence, ensuring that the learned HR dictionary not only satisfies sparsity well, but also has less dimensionality to speed up the sparse decomposition. Then, a variational regularization HSI-SR model combing a spatial sparsity regularization term and a new local spectral similarity preserving term is proposed to integrate the spectral and spatial-contextual information of the HSI. Finally, the experimental results show that the proposed method not only enhances the spatial resolution, but also preserves the spectral information of the HSI well.

The remainder of this paper is organized as follows: in Section 2, we introduce the basic theory of CS. The HR dictionary learning approach with strong sparsity and small coherence and the proposed spatial-spectral-based regularization HSI-SR model are described in Section 3. In Section 4, the experimental results are shown and compared with other approaches. Finally, conclusions are drawn in Section 5.

## 2. The CS Theory

This paper proposes a novel HSI-SR method and a new dictionary learning approach from the perspective of the CS theory. In this section, we will introduce the basic theory of CS. Suppose that LR HSI **Y***_k_* and HR HSI **X***_k_*, where *k* = 1,…,*P*, with *P* being the number of spectral bands, the relationship between **Y***_k_* and **X***_k_* can be modeled as:

(1) $${\text{Y}}_{k}={\text{SHX}}_{k}+\text{v}$$

where **S** is a down-sampling operator, **H** is a blurring operator and **v** is additive noise.

We represent the liner observation matrix **L** = **SH** as the sensing matrix of CS theory. Because the dimension of the **Y***_k_* is less than the dimension of the **X***_k_*, the backward process from **Y***_k_* to **X***_k_* solves an underdetermined equation that cannot provide a unique solution. According to CS theory, in order to correctly recover **X***_k_* from the observation **Y***_k_*, we must satisfy the following two conditions [18]:

(1) Sparsity: Given a column vector **x***_i_* ∈ ℝ*^n^* representing a lexicographically ordered HR image patch, **x***_i_* = **R***_i_***X***_k_*, (*i* = 1,…,*N*), where **R***_i_* is an extracting operator, *N* is the number of HR image patches. Sparsity assumes that **x***_i_* can be sparsely represented by an over-complete dictionary **Φ** ∈ ℝ*^n^*^×^*^t^, i.e.*, **x***_i_* = **Φα***_i_*, where **α***_i_* is sparse representation coefficients and most of them are zero. Thus, it can be written the following *l*_0_-norm optimization problem: $\min{\left\|{\text{x}}_{i}-\Phi \alpha_{i}\right\|}_{2}^{2}$, *s.t.*‖**α***i*‖_0_≤*T*_0_, where *T*_0_ is sparsity constraint parameter.
(2) Incoherence: The coherence between the sensing matrix **L** ∈ ℝ*^m^*^×^*^n^* and the dictionary **Φ** is: $\mu (\text{L},\Phi )=\sqrt{n}\cdot \max_{1\leq p\leq m,1\leq q\leq t}\left|\langle l_{p},\phi_{q}\rangle \right|$, where *l_p_* is the *p*-th row of **L** and *ϕ_q_* is the *q*-th column of **Φ**. There is $\mu (\text{L},\Phi )\in [1,\sqrt{n}]$. The CS theory proves that the coherence μ is smaller, the reconstruction result is better.

## 3. Proposed HSI-SR Method

**Figure 1.**
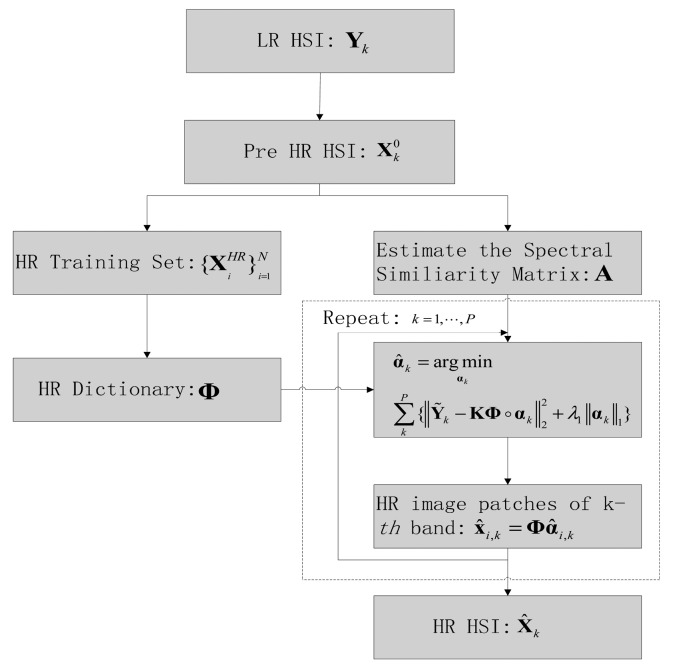
Flowchart of the proposed method.

Different from the traditional SR methods for natural images, the SR methods for HSI not only improves the spatial resolution, but also preserves the spectral information well. In this section, we will present a novel HSI-SR framework from the perspective of CS theory, which introduces a sparsity regularization for recovering the spatial information of HR HSI from the observed LR HSI. Meanwhile, in order to better preserve the spectral information, a spectral regularization based on the similarity of spectral curves is introduced into the HSI-SR model. Moreover, the CS-based HSI-SR methods requires a HR dictionary that can sparsely represent the HR image patches with the linear combination of a few atoms. Here, the HR dictionary is learned by a new dictionary learning method. Therefore, the proposed method contains three parts: (1) learning the HR dictionary with strong sparsity and small coherence; (2) a spatial sparsity regularization term for the HSI-SR model; (3) a new nonlocal spectral similarity preserving term for HSI-SR model. The flowchart of the proposed method is shown in Figure 1.

### 3.1. Learning the HR Dictionary with Strong Sparsity and Small Coherence

As we all know, the choice of dictionary plays a very important role in the field of sparse representation. The CS-based HSI-SR method requires learning a HR dictionary, which is learned from the observed LR image itself instead of the HR image training database. The fundamental notion of these methods is that a large number of similar patches may exist in the same LR image, both within the same scale and across different scales. Pan *et al.* [22] have shown that a dictionary using the pre-HR image as the training sample is better than that trained using the HR image training database. In this section, a new CS-based dictionary learning method for HSI-SR problem is proposed. Like [22], the HR dictionary is trained from pre-HR HSI instead of panchromatic images or HR HSI training database. The pre-HR HSI is formed from the observed LR HSI by using the bicubic interpolation.

Inspired by two conditions of CS theory in the Section 2, this paper learns a HR dictionary with strong sparsity and small coherence. For generating the HR training set **X***^HR^*, concatenating all the database vectors column-wise into a matrix **X***^HR^* = {**x**_1_,…,**x***_i_*,…,**x***_N_*} ∈ ℝ*^n^*^×^*^N^*, where **x***_i_* is the column vector representing a lexicographically ordered HR image patch, which samples from pre-HR HSI and *N* is the number of training image patches.

In order to simultaneously satisfy two conditions of CS theory, our dictionary learning task can be modeled as:

(2) $$\min_{\left\{\Phi ,\alpha \right\}}{\left\|{\text{X}}^{HR}-\Phi \alpha \right\|}_{F}^{2},\;s.t.{\left\|\alpha \right\|}_{0}\leq T_{0},\mu \left(\text{L},\Phi \right)\leq T_{1}$$

where **α** ∈ ℝ*^t^*^×^*^N^* is a matrix containing all the corresponding sparse representation coefficients **α***_i_, T*_0_ is sparsity constraint parameter and *T*_1_ is the threshold of the coherence.

The CS-based dictionary learning approach makes full use of the strong sparsity and incoherence of the CS theory. This strategy makes the coherence between learned dictionary and sensing matrix become smaller. Furthermore, instead of a fixed number of dictionary atoms, our method can reduce the dimensionality of the dictionary according to the threshold of coherence, which speeds up the sparse decomposition. The algorithm of solving model (2) is summarized in Algorithm 1.



**Algorithm 1** CS-based Dictionary Learning Algorithm
**Input**: The LR HSI 
${\left\{{\text{Y}}_{k}\right\}}_{k=1}^{P}$, the training set 
${\left\{{\text{X}}_{i}^{HR}\right\}}_{i=1}^{N}$ and the linear observation matrix **L**.(1) **Initialize** the dictionary **Φ**^0^ ∈ ℝ*^m^*^×^*^t^* randomly.(2) **Repeat** the following steps and increase *r* by 1:  (2.1) Fixing the dictionary, we use OMP algorithm to obtain sparse coefficients **α***^r^* in Equation (2).  (2.2) We use the update stage of K-SVD method to update each column of the dictionary and obtain dictionary **Φ***^r^*.  (2.3) Compute the coherence matrix 
$\text{T}\in {\text{R}}^{m\times t}:\text{T}=\sqrt{n}{\left|\langle l_{p},\phi_{q}\rangle \right|}_{1\leq p\leq m,1\leq q\leq t}$, then find the maximum of each column in matrix T to obtain the vector 
${\left\{{\hat{\tau }}_{q}\right\}}_{q=1}^{t}\in {\text{ℝ}}^{t}$.  (2.4) If exiting *τ̂_q_* > *T*_1_, then delete the corresponding atom of the dictionary **Φ***^r^* to obtain a new dictionary **Φ**^*r*+1^ ∈ ℝ^*m*×*t*′^(*n* ≤ *t*′ ≤ *t*), Otherwise **Φ**^*r*+1^ = **Φ***^r^*.**Until** the change in 
${\left\|{\text{X}}^{HR}-\Phi^{r+1}\alpha^{r}\right\|}_{F}^{2}$ is enough small, stop.Output: The learned dictionary **Φ**^*r*+1^.


### 3.2. Spatial Sparsity Regularization Term

The HSI-SR method aims at recovering the HR HSI **X***_k_* with high spatial resolution and less spectral distortions from the observed LR HSI **Y***_k_*. As we all know, the CS-based HSI-SR methods are difficult to process the high-dimensional signals directly, especially for the case of the HR dictionary **Φ** requires learning. Therefore, each band of the LR HSI **Y***_k_* is divided into image patch set {**y***_i,k_*} (size: $\sqrt{m}\times \sqrt{m}$) with overlap instead of processing the whole image directly, where **y***_i,k_* denotes the *i*-th image patch in the *k*-th band of observed LR HSI.

According to the LR dictionary **LΦ**, we seek the sparse representation coefficient **α***_i,k_* for each LR image patch, and then employ the coefficient **α***_i,k_* multiplying with the HR dictionary **Φ** to generate the corresponding HR image patch **x***_i,k_* (size: $\sqrt{n}\times \sqrt{n}$), which can be written as:

(3) $${\text{x}}_{i,k}=\Phi \alpha_{i,k}$$

where **x***_i.k_* denotes the *i*-th image patch in the *k*-th band of the reconstructed HR HSI.

Then, by averaging all of these patches of the image patch set {**x***_i.k_*}, the reconstructed HR HSI **X***_k_* can be written as:

(4) $${\text{X}}_{k}={\left(\overset{N}{\underset{i=1}{\text{∑}}}{\text{R}}_{i}^{T}{\text{R}}_{i}\right)}^{†}\overset{N}{\underset{i=1}{\text{∑}}}\left({\text{R}}_{i}^{T}{\text{x}}_{i,k}\right)$$

where † denotes the pseudo-inverse. For the convenience of expression, we define the following operator “∘” as:

(5) $${\text{X}}_{k}=\Phi \circ \alpha_{k}={\left(\overset{N}{\underset{i=1}{\text{∑}}}{\text{R}}_{i}^{T}{\text{R}}_{i}\right)}^{†}\overset{N}{\underset{i=1}{\text{∑}}}\left({\text{R}}_{i}^{T}\alpha_{i,k}\right)$$

where **α***_k_* is the concatenation of the **α***_i,k_* of the *k*-th band.

Since Equation (1) is an underdetermined equation, we cannot obtain a unique solution. In order to find a unique or optimal solution, some constraints can be used to regularize Equation (1). Therefore, according to CS theory, sparsity regularization is constrained on the spatial information of HSI to ensure Equation (1) has a unique solution. Then, combined with the Equation (5), the Equation (1) can be written as:

(6) $${\hat{\alpha }}_{k}=\underset{\alpha_{k}}{\arg\min}\overset{P}{\underset{k=1}{\text{∑}}}\left\{{\left\|{\text{Y}}_{k}-\text{L}\Phi \circ \alpha_{k}\right\|}_{2}^{2}+\lambda_{1}{\left\|\alpha_{k}\right\|}_{0}\right\}$$

where λ_1_ is a balancing parameter determined by cross validation. Although the *l*_0_-norm optimization problem is NP-hard, recent results indicate that as long as the coefficient **α***_i,k_* is sparse enough, they are often replaced by *l*_1_-norm to solve the optimization problem [23]. Therefore, Equation (6) can be rewritten as follows:

(7) $${\hat{\alpha }}_{k}=\underset{\alpha_{k}}{\arg\min}\overset{P}{\underset{k=1}{\text{∑}}}\left\{{\left\|{\text{Y}}_{k}-\text{L}\Phi \circ \alpha_{k}\right\|}_{2}^{2}+\lambda_{1}{\left\|\alpha_{k}\right\|}_{1}\right\}$$

As we all know that the HSI is a three-dimensional data cube, which contains the spatial information and spectral information. Besides introducing the sparsity regularization into HSI-SR model to constrain the spatial information, we will discuss how to better preserve the spectral information in the next subsection.

### 3.3. New Nonlocal Spectral Similarity Preserving Term

**Figure 2.**
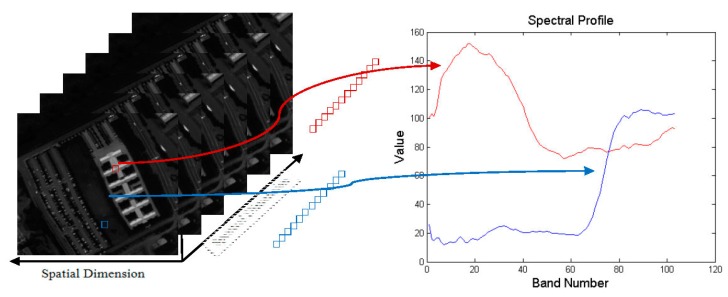
Schematic view of the HSI data. Left: A three dimensional HSI data. Right: Reflected spectral curve at the corresponding pixels of each band in the HSI.

If we use Equation (7) directly to reconstruct each HSI band individually, the spectral information will be ignored. The spectral information is a set of reflectance values consisting of all spectral curves at the corresponding pixels of each band in the HSI (as shown in Figure 2). The same scene of the HSI contains many similar materials that have similar spectral curves, such as building, road and meadow, *etc.* We use the similarity of spectral curves to regularize the HSI-SR model, which is very helpful in preserving the spectral information and improving the quality of reconstructed HSI.

To find the similar pixels of a given pixel in the whole image, we utilize a new similarity measure [24], which integrates the spectral and spatial-contextual information in the HSI. Given that a spatial window *SN*(*x_ij_*) (size: *ω* × *ω*) with central pixel *x_ij_*, where *ω* is an odd positive integer, *SN*(*x_ij_*) is a *ω* × *ω* -pixel set consisting of central pixel *x_ij_* and its spatial neighbor pixels and can be defined as follows:

(8) $$SN\left(x_{ij}\right)=\left\{x_{pq}:p=i-r,\cdots ,i,\cdots i+r;q=j-r,\cdots ,j,\cdots j+r\right\}$$

where *r* = (*ω*−1)/2. When the spatial window falls outside the image, a periodic extension of the image is assumed.

**Figure 3.**
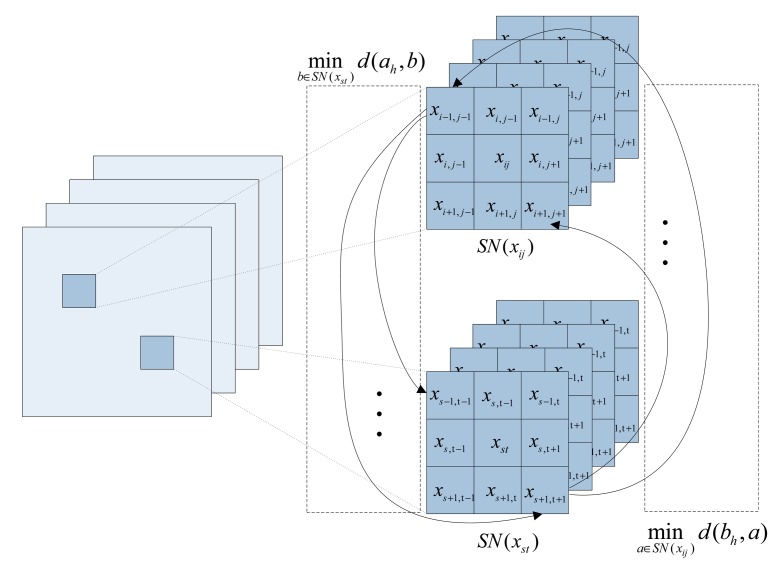
Flowchart of concrete process of calculating IPD with the 3 × 3 spatial window.

The new similarity measure (named image patch distance (IPD)) between the observation pixels *x_ij_* and *x_st_* is defined as:

(9) $$\begin{array}{l} d_{\text{IPD}}\left(x_{ij},x_{st}\right)=d\left(SN\left(x_{ij}\right),SN\left(x_{st}\right)\right)\\ \;=\overset{\omega \times \omega }{\underset{l=1}{\text{∑}}}\max\left(\underset{b\in SN\left(x_{st}\right)}{\min}d\left(a_{h},b\right),\underset{a\in SN\left(x_{ij}\right)}{\min}d\left(b_{h},a\right)\right) \end{array}$$

where *d*(*a*,*b*) is a spectral similarity function, *a_h_* and *b_h_* are the *_h_-th* element of the spatial windows *SN*(*x_ij_*) and *SN*(*x_st_*), respectively. In order to find the similar pixel *x_st_* with the observation pixel *x_ij_*, the concrete process of calculating IPD is shown in Figure 3. For simplicity, the minimum value of *ω* (*i.e., ω* = 3) is chosen. From Figure 3, we can see that the same coordinates of the pixels in each band of the HSI constitute the spectral vector. Thus, the IPD reflects the similarity of spectral curves in the HSI.

According to Equation (9), we can obtain a column vector ${\text{Q}}_{ij}^{l}$ that stacks first the *L* most similar pixels $x_{st}^{l}$ as relevant neighbors of the pixel *x_ij_, i.e.*, ${\text{Q}}_{ij}^{l}={\left\{x_{st}^{1},\cdots ,x_{st}^{l},\cdots ,x_{st}^{L}\right\}}^{T}$. Therefore, the value of the pixel *x_ij_* can be regarded as the weighted average of all points in the ${\text{Q}}_{ij}^{l}$ [25] as follows:

(10) $$x_{ij}=\overset{L}{\underset{l=1}{\text{∑}}}\omega_{\left(ij\right)\left(st\right)}^{l}x_{st}^{l}$$

where the weight $\omega_{\left(ij\right)\left(st\right)}^{l}$ represents the similarity between the pixels *x_ij_* and $x_{st}^{l}$, which can be calculated by:

(11) $$\omega_{\left(ij\right)\left(st\right)}^{l}=\frac{1}{\text{W}}\exp\left(-d_{\text{IPD}}\left(x_{ij},x_{st}^{l}\right)/h\right)$$

where *h* is a pre-determined scalar and W is the normalization factor. Let ${\text{w}}_{ij}^{l}$ be a row vector containing all the weights $\omega_{\left(ij\right)\left(st\right)}^{l}$, *i.e.*, ${\text{w}}_{ij}^{l}=\left\{\omega_{\left(ij\right)\left(st\right)}^{1},\cdots ,\omega_{\left(ij\right)\left(st\right)}^{l},\cdots ,\omega_{\left(ij\right)\left(st\right)}^{L}\right\}$. Thus, Equation (10) can be reformulated in a vector-matrix form:

(12) $$x_{ij}={\text{w}}_{ij}^{l}{\text{Q}}_{ij}^{l}$$

Using Equation (12), we can define a nonlocal spectral similarity preserving term expressed by:

(13) $$R\left({\text{X}}_{k}\right)={\underset{x_{ij}\in {\text{X}}_{k}}{\text{∑}}\left\|x_{ij}-{\text{w}}_{ij}^{l}{\text{Q}}_{ij}^{l}\right\|}_{2}^{2}$$

For the convenience of expression, all the pixels of HSI **X***_k_* are stacked into a vector in lexicographic order. Let us denote *x_u_* and *x_v_* be the *u-th* and *v-th* pixels of the vector, respectively. Computing the similarity of each pixel-pair according to the Equation (11), we then can define the weighting matrix **A**, whose elements A(*u,v*) are defined by:

(14) $$\text{A}\left(u,v\right)=\{\begin{array}{ll} \omega_{uv}^{l}, & \text{if}\;x_{v}\in {\text{Q}}_{u}^{l},\omega_{uv}^{l}\in {\text{w}}_{u}^{l}\\ 0, & \text{otherwise} \end{array}$$

Thus, Equation (13) can be written in a vector-matrix form:

(15) $$R\left({\text{X}}_{k}\right)={\left\|{\text{X}}_{k}-{\text{AX}}_{k}\right\|}_{2}^{2}$$

Combining Equations (7) and (15), the final proposed model can be written as a concise form according to the Equation (5):

(16) $$\arg\min_{\left\{\alpha_{k}\right\}}\overset{P}{\underset{k=1}{\text{∑}}}\left\{{\left\|{\text{Y}}_{k}-\text{L}\Phi \circ \alpha_{k}\right\|}_{2}^{2}+\lambda_{1}{\left\|\alpha_{k}\right\|}_{1}+\lambda_{2}{\left\|(\text{I}-\text{A})\Phi \circ \alpha_{k}\right\|}_{2}^{2}\right\}$$

where *λ*_2_ is a balancing parameter and **I** is the identity matrix. By letting ${\tilde{\text{Y}}}_{k}=\left[\begin{array}{c} {\text{Y}}_{k}\\ 0 \end{array}\right]$, $\text{K}=\left[\begin{array}{l} \text{L}\\ \lambda_{2}(\text{I}-\text{A}) \end{array}\right]$, Equation (16) can be rewritten as:

(17) $$\arg\min_{\left\{\alpha_{k}\right\}}\overset{P}{\underset{k=1}{\text{∑}}}\left\{{\left\|{\tilde{\text{Y}}}_{k}-\text{K}\Phi \circ \alpha_{k}\right\|}_{2}^{2}+\lambda_{1}{\left\|\alpha_{k}\right\|}_{1}\right\}$$

Here, we use iterative shrinkage algorithm [26] to seek the solution of this equation.

## 4. Experimental Results

The experimental results are presented below based on analysis of two kinds of HSI datasets. One is a simulated experiment for two different hyperspectral remote sensing datasets. They are Pavia University (PaviaU) dataset and Pavia Center (PaviaC) dataset, respectively. Another kind is a real experiment, which was conducted on two different HSI datasets that were collected by the Hyperspec VNIR hyperspectral imaging spectrometer with a spectral range from 400 nm to 1000 nm. One is an indoor scene over letter paper and the other is an outdoor scene over Fountain Square of Nanjing University of Science and Technology (FS-NUST).

In order to evaluate the proposed HSI-SR method, we use the following three quality indexes: average peak signal noise ratio (A-PSNR), average structural similarity (A-SSIM) and spectral angle mapper (SAM). For the HSI dataset, we firstly estimate the PSNR and SSIM of every spectral band individually. Then, the A-PNSR and A-SSIM are estimated by averaging over all the spectral bands. SAM reflects the spectral distortion by the absolute angles between two spectral vectors constructed from each pixel of the original and reconstructed HSI, respectively, and the measure SAM is computed by averaging over the whole image. For an ideal reconstructed image, the value of SAM should be zero.

### 4.1. Simulation Experiments

The PaviaU and PaviaC datasets were collected by the Reflective Optics System Imaging Spectrometer sensor in the framework of the HySens project managed by the German Aerospace Center [27]. The images have 115 spectral bands with spectral ranges from 430 nm to 860 nm. Twelve channels of the PaviaU dataset were removed due to noise. The remaining 103 spectral bands (of size 210 × 210) were processed. For another HSI dataset, thirteen channels of the PaviaC dataset were removed due to noise. The remaining 102 spectral bands (of size 210 × 210) were processed.

In the process of simulation experiments, the LR HSI datasets are obtained from the original HSI datasets by convolving with a 3 × 3 Gaussian kernel of standard deviation 1.6, down-sampling by a factor of 3 and without noise. The results are compared with the reconstructed images obtained by bicubic interpolation method (implemented with MATLAB R2012a), principal component analysis (PCA) image fusion method [11], a discrete wavelet transform (WT) image fusion method [13] and a variational model for P + XS image fusion [15] (the software and documentation is available online at: www.math.ucla.edu/∼wittman/pansharpening), and sparse representation-based SR (SR-SR) method [20]. In order to conduct the experiments of image fusion, one HR panchromatic image was created by spectrally integrating over all spectral bands of the original HSI [28].

#### 4.1.1. PaviaU Dataset

The first simulation experiment was divided into two phases. In the CS-based dictionary learning phase, we randomly sample 10,000 training image patches (of size 9 × 9) from the pre-HR HSI dataset. The corresponding parameters are set as following: the number of the atoms of the dictionary **Φ**^0^ is initialized to 500 and the threshold of the coherence *T*_1_ is set as 1.8. In the SR reconstruction phase, we firstly use pre-HR HSI to estimate the weights *ω_st_* of the spectral similarity, where *d*(*a*,*b*) in the Equation (9) is calculated by the Euclidean Distance. Then, according to the matrix of the weights **A** and the learned dictionary **Φ**, we can obtain the sparse representation coefficients *α_k_*. The corresponding parameters λ_1_ and λ_2_ are set as 0.025 and 0.04 by cross validation, respectively. In addition, we divide the LR HSI into 3 × 3 image patches with one pixel width overlap between adjacent patches.

**Figure 4.**
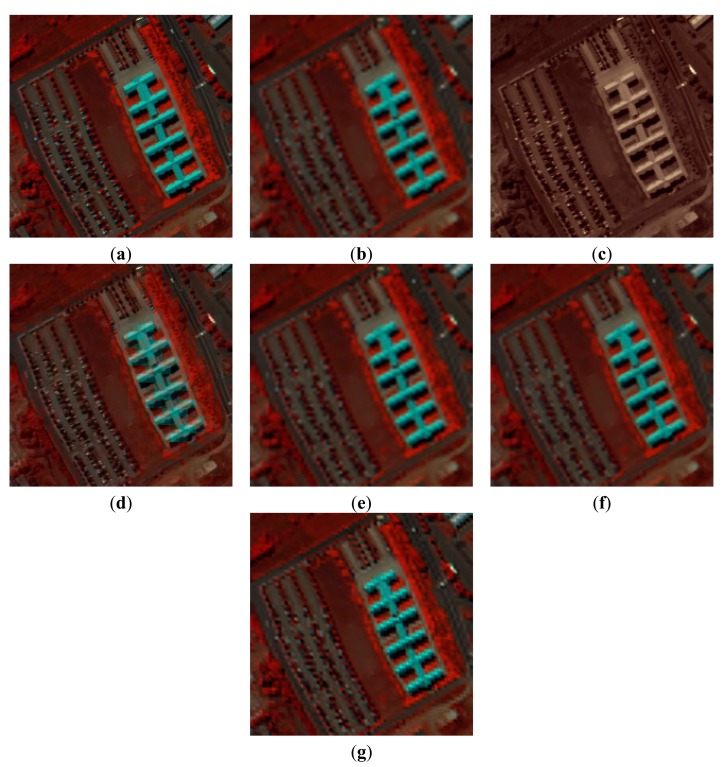
Experimental results of PaviaU HSI (color composite of R: 80, G: 28, B: 9). (**a**) the false color image of original HSI; (**b**) bicubic interpolation; (**c**) PCA fusion method; (**d**) WT fusion method; (**e**) P + XS fusion method; (**f**) SR-SR method; (**g**) the proposed method.

The next issue is the evaluation and analysis of the reconstructed images. The visual results of the PaviaU HSI dataset with different SR reconstruction methods are shown in Figure 4. For the purpose of visualization by a human observer, we choose the 80th, 28th and 9th spectral band of the PaviaU dataset as the R, G and B channels of the color images in Figure 4, respectively. The false color image of the original HSI is shown in Figure 4a. We can see that the result of bicubic interpolation method shown in Figure 4b will produce noticeable blurring and ringing artifacts along the edges and corners. Figure 4c,d illustrate the results of the PCA image fusion and WT image fusion methods, respectively. They preserve the structural information, but they fail in recovering the color information, which reflects poor performance in preserving the spectral information. A variational model for P + XS image fusion shown in Figure 4e can preserve the spectral information better than the above two image fusion methods. From Figure 4f reconstructed by the SR-SR method, we can see that it can achieve better spatial-spectral information recovery than the P + XS image fusion method. On the whole, the result of the proposed method shown in Figure 4f is the best of all the methods compared with the false color image of the original HSI.

**Figure 5.**
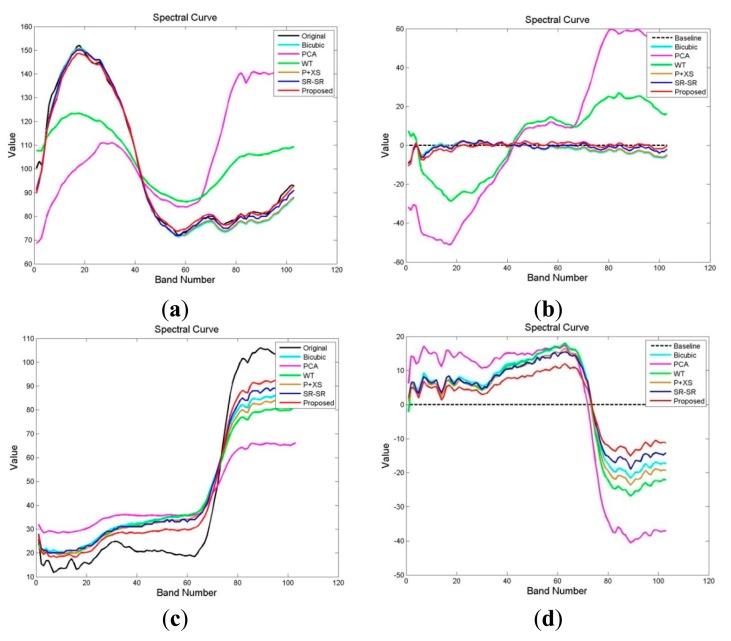
Spectral curves of two typical pixels, one is metal material (the coordinate located at (72,129)), the other is meadow material (the coordinate located at (127,173)). (**a**) the spectral curves of the metal material; (**b**) the pixel curves of the metal material in difference HSI; (**c**) the spectral curves of the meadow material; (**d**) the pixel curves of the meadow material in difference HSI.

In order to further compare the spectral preservation information with the abovementioned methods, we select two typical pixels (one is metal material (the coordinate located at (72,129)), and the other is meadow material (the coordinate located at (173,127))), and draw the spectral curves of the different experimentalresults. The spectral curves of HSI reconstructed by different methods are shown in Figure 5a,c, respectively. The closer the reconstructed spectral curve is to the original spectral curve, the better the result is. Figure 5b,d show the abovementioned two pixel curves of difference HSI obtained by subtracting the restoration results from the noise-free spectrum. The closer the pixel curves of the difference HSI is to the baseline, the better the result is. As shown in Figure 5, we can clearly see that the PCA fusion and WT fusion methods are very poor in preserving the spectral information in two pixels. The spectral curves of the bicubic interpolation, P + XS fusion and SR-SR methods change a little. Compared with these methods, the spectral curves reconstructed by the proposed method are the best of all reconstructed spectral curves.

Table 1 shows the comparison results of A-PSNR, A-SSIM and SAM of the reconstructed HSI by different methods in Figure 4. We can see that our proposed method outperforms the other methods in spatial-spectral information reconstruction of the HSI dataset.

#### 4.1.2. PaviaC Dataset

**Figure 6.**
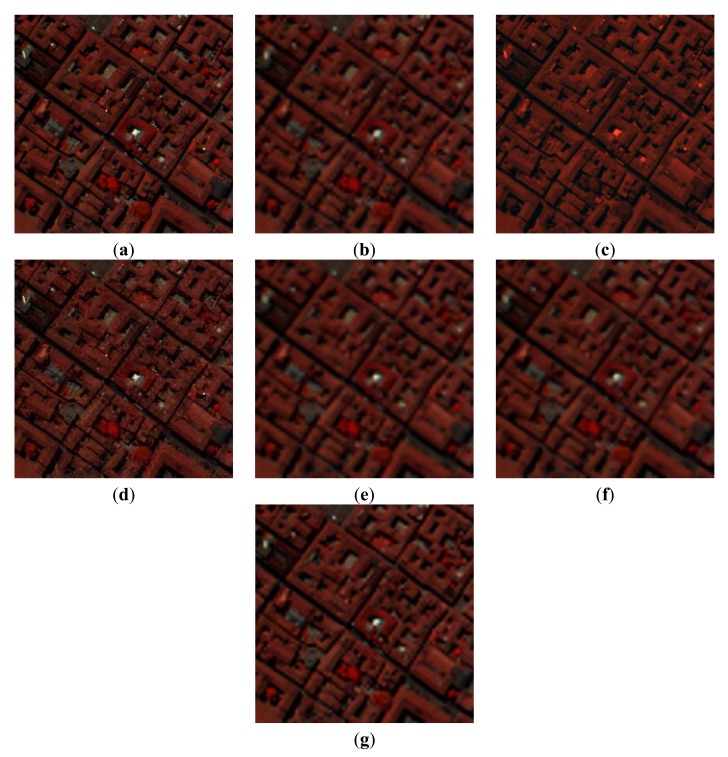
Experimental results of PaviaU HSI (color composite of R: 80, G: 28, B: 9). (**a**) the false color image of the original HSI; (**b**) bicubic interpolation; (**c**) PCA fusion method; (**d**) WT fusion method; (**e**) P + XS fusion method; (**f**) SR-SR method; (**g**) the proposed method.

The second simulation experiment was conducted on the PaviaC dataset. The parameters of two phases are the same as the first simulation experiment. Figure 6a shows the original false color image consisting the 80th (R), 28th (G) and 9th (B) spectral band. Figure 6b–g show the different result images reconstructed by bicubic interpolation method, PCA image fusion method, WT image fusion method, a variational model for P + XS image fusion, SR-SR method and proposed method, respectively. From the Figure 6, we can see that Figure 6g reconstructed by the proposed method is closest to the false color image of original HSI.

Table 2 shows the comparison results of A-PSNR, A-SSIM and SAM of the reconstructed HSI by the different methods in Figure 6. Like Table 1, the proposed method performs the best in the different quality indexes.

### 4.2. Real Experiments

In this section, we describe and analyze the experimental results on two real HSI datasets. The first HSI dataset of an indoor scene over letter paper has 60 spectral bands (60 × 120 size and without noisy bands), with spectral range from 400 nm to 1000 nm, and a 7.88 nm spectral interval. The second HSI dataset of an outdoor scene over FS-NUST has 110 spectral bands (86 × 86 size and without noisy bands), with spectral range from 400 nm to 1000 nm, and a 4.73 nm spectral interval.

In the process of real experiments, the blurring operator **H** in the model (1) is unknown. In order to reconstruct the HR HSI dataset, we only focus on the situation where the blur kernel is the Dirac delta function, the scaling factor is 3 and the noise **v** is zero. Because these are not real HR panchromatic images, we cannot conduct the image fusion experiments. We compare the results of the proposed method with those of bicubic interpolation method and SR-SR method via visual evaluation.

#### 4.2.1. Indoors Experiment

**Figure 7.**
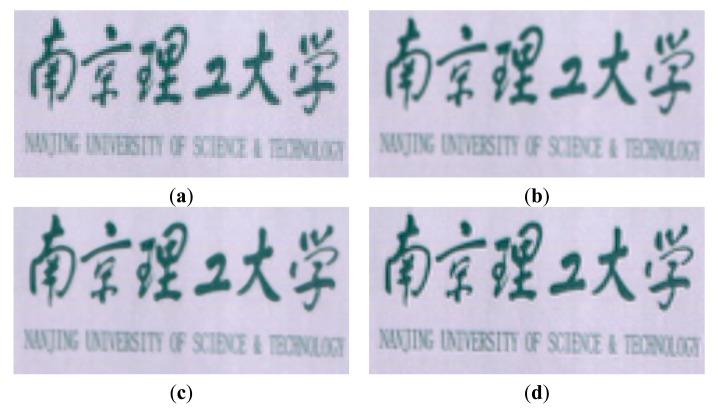
Experimental results of Letter paper HSI dataset (color composite of R: 31, G: 20, B: 8). (**a**) original LR HSI; (**b**) bicubic interpolation method; (**c**) SR-SR method; (**d**) the proposed method.

**Figure 8.**
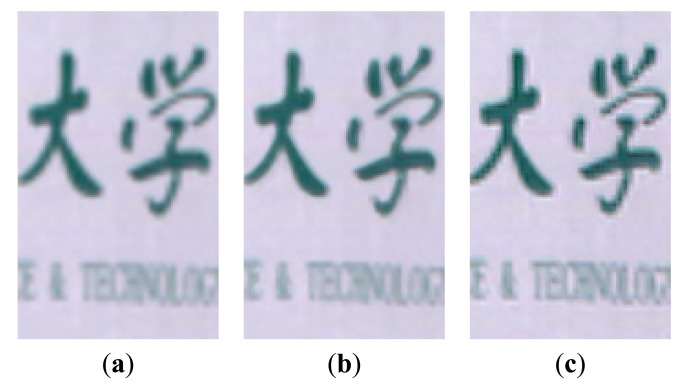
The cropped portions of the Figure 5b–d (color composite of R: 31, G: 20, B: 8). (**a**) bicubic interpolation method; (**b**) SR-SR method; (**c**) the proposed method.

The HR dictionary is learned from the pre-HR HSI dataset that is generated by using the bicubic interpolation from the LR letter paper HSI dataset. The corresponding parameters λ_1_ and λ_2_ are set as 0.015 and 0.02, respectively. Figure 7a shows a color image of the original LR letter paper HSI dataset, with a color composite of the 31th (R), 20th (G) and 8th (B) spectral bands. Figure 7b–d show the results of the bicubic interpolation method, SR-SR method and our proposed method, respectively. In Figure 8, we show some cropped portions of the Figure 7b–d. From these figures, we can see that the proposed method produces sharper edges and clearer details than the bicubic interpolation method and SR-SR method.

#### 4.2.2. Outdoors Experiment

**Figure 9.**
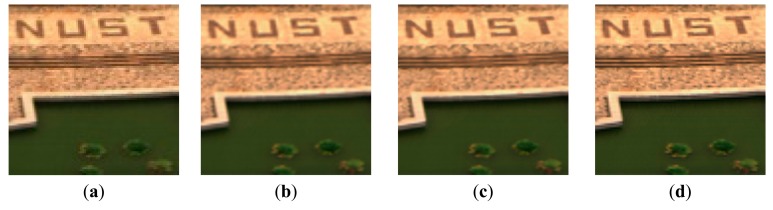
Experimental results of FS-NUST HSI dataset (color composite of R: 52, G: 32, B: 13). (**a**) original LR HSI; (**b**) bicubic interpolation method; (**c**) SR-SR method; (**d**) the proposed method.

The HR dictionary is learned from pre-HR HSI dataset that is generated by using the bicubic interpolation from the FS-NUST HSI dataset. The corresponding parameters λ_1_ and λ_2_ are set as 0.025 and 0.02, respectively. A color image of original LR FS-NUST HSI dataset with a color composite of the 52th (R), 32th (G) and13th (B) spectral bands is shown in Figure 9a. Figure 9b–d show the results of the bicubic interpolation method, SR-SR method and the proposed method, respectively.

**Figure 10.**
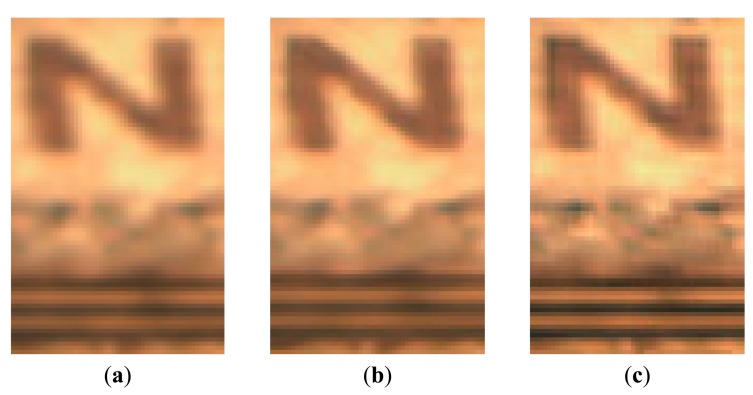
The cropped portions of the Figure 7b–d (color composite of R: 52, G: 32, B: 13). (**a**) bicubic interpolation method; (**b**) SR-SR method; (**c**) the proposed method.

Some cropped portions of Figure 9b–d are shown in Figure 10. From these reconstructed results, we can see that the proposed method produces sharper edges and clearer details than bicubic interpolation and SR-SR method.

## 5. Conclusions

In this paper, a novel HSI-SR method using spatial-spectral information of the HSI and a CS-based new dictionary learning approach are proposed. Based on the LR HSI generation model, we referred to the HSI-SR problem as an ill-posed inverse problem. In order to better reconstruct spatial-spectral information of the HR HSI, spatial sparsity regularization and spectral similarity regularization are employed to address this ill-posed inverse problem. Moreover, we make full use of two conditions of CS theory to construct the HR dictionary for better performance in the HSI-SR problem. The experimental results on simulated and real HSI datasets suggest that the proposed method can achieve competitive spatial quality compared to the other well-known methods and preserve spectral information well.

One drawback of the proposed method is that this method takes more CPU time than the other methods, since it needs to process hundreds of channels in the HSI by a band-by-band manner and search for the similar pixels with the observation pixel in the HSI by IPD. In future work, we will reduce the dimensions of the HSI and explore the use of parallel-based many-core algorithms to speed up the proposed method.

## Figures and Tables

**Table 1.** Comparison Results of A-PSNR, A-SSIM and SAM of the Different Methods.

**HSI**	**Measures**	**Bicubic Method**	**PCA Fusion**	**WT Fusion**	**P**+**XS Fusion**	**SR-SR Method**	**Proposed Method**
PaviaU	A-PSNR	25.7229	24.3450	27.0231	26.1277	26.7629	**27.9708**
	A-SSIM	0.7172	0.7608	0.7776	0.7397	0.7713	**0.8017**
	SAM	0.1025	0.2675	0.1598	0.1028	0.0951	**0.0866**

**Table 2.** Comparison results of A-PSNR, A-SSIM and SAM of the different methods.

**HSI**	**Measures**	**Bicubic Method**	**PCA Fusion**	**WT Fusion**	**P**+**XS Fusion**	**SR-SR Method**	**Proposed Method**
PaviaC	A-PSNR	26.4295	28.6203	28.8406	29.3209	28.6839	**30.8177**
	A-SSIM	0.6843	0.8475	0.8251	0.8025	0.7697	**0.8630**
	SAM	0.1084	0.1822	0.1667	0.0969	0.0992	**0.0866**
